# Skeletal Muscle Insulin Resistance in a Novel Fetal Growth Restriction Model

**DOI:** 10.3390/pediatric15010006

**Published:** 2023-01-16

**Authors:** Kazuhide Tokita, Hiromichi Shoji, Yoshiteru Arai, Kentaro Awata, Irena Santosa, Yayoi Murano, Toshiaki Shimizu

**Affiliations:** 1 Department of Pediatrics and Adolescent Medicine, Juntendo University Graduate School of Medicine, Hongo, Bunkyo-ku, Tokyo 113-8421, Japan; 2 Department of Pediatrics, Juntendo University Faculty of Medicine, Hongo, Bunkyo-ku, Tokyo 113-8421, Japan

**Keywords:** fetal growth restriction, insulin resistance, skeletal muscle, ameroid constrictor, insulin signaling, GLUT4, insulin signaling

## Abstract

The abnormal fetal environment exerts long-term effects on skeletal muscle, and fetal growth restriction (FGR) is associated with insulin resistance in adulthood. In this study, we examined insulin resistance in early adulthood and insulin signaling in skeletal muscle using a novel FGR rat model. Ameroid constrictors (AC) were placed on the bilateral uterine and ovarian arteries of rats on day 17 of gestation; placebo surgery was performed on the control group. We measured body weight at birth, 4, 8, and 12 weeks of age and performed oral glucose tolerance tests at 8 and 12 weeks. Rats were dissected at 12 weeks of age. We examined the mRNA and protein expression of insulin signaling pathway molecules in skeletal muscle. FGR rats had a significantly lower birth weight than control rats (*p* = 0.002). At 12 weeks of age, the incremental area under the curve of blood glucose was significantly higher, and GLUT4 mRNA and protein expression in soleus muscle was significantly lower in the FGR group than in the control group. Reduced placental blood flow in the AC-attached FGR group caused insulin resistance and altered insulin signaling in skeletal muscles. Therefore, FGR causes skeletal muscle insulin resistance in early adulthood.

## 1. Introduction

Fetal growth restriction (FGR) describes a situation in which the fetus does not grow to its expected biological potential in the uterus [1]. Numerous studies based on animals and humans suggest an association between FGR and lifestyle-related diseases such as type 2 diabetes (T2DM), cardiovascular disease, and hypertension in adults [2,3,4]. Recently, the prevalence of low-birth-weight infants due to FGR has been increasing in Japan [5]. Fetal growth is dependent on the continuous transfer of oxygen and nutrients across the placenta from the mother. The common risk factors of FGR due to placental insufficiency are reduced placental blood flow (due to advanced maternal age, hypertension, preeclampsia, etc.) and malnutrition during pregnancy [1]. Infants with reduced placental blood flow during the fetal period have been reported to experience various complications later in life [6,7].

Insulin resistance is one of the main mechanisms of lifestyle-related diseases. Skeletal muscle is the primary location for insulin-stimulated glucose uptake, accounting for up to 70% of whole-body glucose disposal [8]. Such glucose disposal is a key regulator of whole-body energy metabolism [9]. Upon binding to its receptor, insulin facilitates glucose uptake in skeletal muscles primarily through glucose transporters, such as glucose transporter isoform 4 (GLUT4). In this process, a distinct signaling cascade is activated that includes multiple enzymes such as the phosphoinositide 3-kinase (PI3K)/protein kinase B (also known as Akt) pathway [10,11].

Any abnormalities in insulin signaling pathways may cause a reduction in insulin sensitivity. In general, qualitative changes in fat cells and the attenuation of insulin action due to inflammation are the main causes of insulin resistance. However, recent research has found that low lean body mass might contribute to insulin resistance in underweight adult women [12]. Another study found that skeletal muscle mass in adulthood was lower in FGR infants [13]. Infants with low birth weight are known to have a higher risk of developing T2DM [14,15].

**Figure 1:**
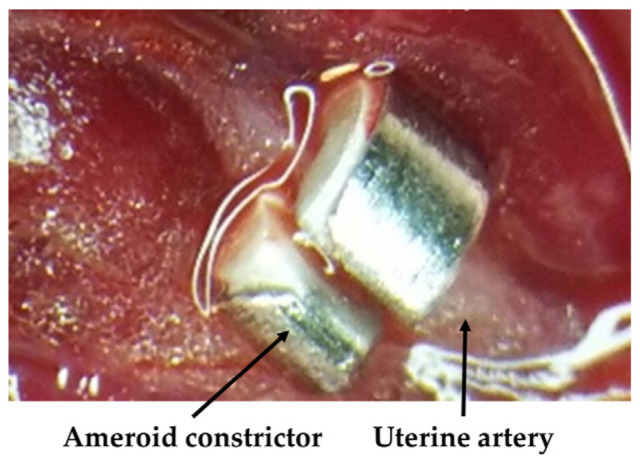
Ameroid constrictor attachment to the uterine artery for reduced placental flow in the fetal growth restriction (FGR) rat model.

Several studies reported various reduced placental flow FGR models primarily using the complete ligation of the bilateral uterine arteries with silk threads [16,17,18,19]. However, it is suggested that in this ligation model, most of the fetal rats proximal to the ligation site will die, and fetal rats far from the ligation site will not experience FGR because of the blood flow from the bilateral ovarian arteries. Therefore, in the present study, we constructed a novel FGR rat model using ameroid constrictors (AC) that have titanium on the outer wall and are composed of C-shaped casein with a notch and a center hole inside which gradually narrows upon absorbing water (Figure 1) [20]. Ameroid constrictors were attached to the bilateral uterine arteries, which reduced the lumen of the blood vessel to a specific size by absorbing water, in turn reducing placental blood flow.

We hypothesized that skeletal muscle degeneration due to FGR and abnormal insulin signaling would lead to insulin resistance without obesity in young offspring. Thus, we planned animal experiments using the novel FGR rat model with reduced placental blood flow by AC attachment.

## 2. Materials and Methods

### 2.1. Animals and Experimental Design

Female Sprague Dawley rats at gestational day (GD)-11 were purchased from Sankyo Labo Service Corporation, Inc. (Tokyo, Japan) and housed in individual cages in the same room at 24–25 °C and 60% relative humidity under a 12:12 h light-dark cycle with free access to food and water at the Juntendo University Animal Care Facility (Tokyo, Japan). In the FGR model rats, we induced anesthesia at GD-17 (equivalent to 20–25 weeks of pregnancy in humans [21]) with 2–2.5% isoflurane and maintained isoflurane at 2% for attachment of four ACs to the bilateral uterine and ovarian arteries (Figure 1) [20]. The AC contains casein and is covered with a wide titanium ring approximately 3.0 mm in diameter and 1.3 mm in width, with a notch and a hole in the center that is 0.4 mm in diameter (SW-MICE-0.4-SS, Tokyo Instruments Inc., Tokyo, Japan). For the control group, placebo operations were performed on the same day. The rats from both groups recovered within an hour after surgery. Food and water were provided ad libitum after recovery. On GD-22, pups were spontaneously delivered, and six male pups were randomly chosen from each group. In this study, only male pups were used to avoid the potential confounding factors of sex and hormones. Up to the weaning period, FGR pups were breastfed by FGR dams, and control pups were fed by control dams. The study protocol was approved by the Animal Care Committee of Juntendo University (1455).

We measured body weight at birth, 4, 8, and 12 weeks of age, and an oral glucose tolerance test (OGTT) was performed at 8 and 12 weeks of age. Dissection was performed at 12 weeks of age; anesthesia was induced with 2–2.5% isoflurane to reduce pain before the dissection. Organ perfusions were performed via the aorta with saline to remove red blood cells. After removing red blood cells, the soleus muscle of the lower limbs was harvested, and portions immersed in RNA later liquid for polymerase chain reaction analysis and stored in liquid nitrogen for western blot analysis; both samples were then stored at −80 °C for further analysis.

### 2.2. Measurement of Glucose Metabolic Indices

Glucose metabolism was assessed using an OGTT. The rats were fasted for 16 h before the test. While performing the OGTT, the water supply was stopped; then, body weight and fasting blood glucose levels were measured. After the initial blood draw, a 2 g/kg glucose solution was administered by oral gavage. Blood glucose levels were measured from blood drawn from the tail vein at 15, 30, 60, 90, and 120 min after glucose administration. The procedure was performed without sedation. Blood glucose levels were measured using Precision Xceed (cat. No 71085-80; ABBOTT Japan, Chiba, Japan), and the incremental areas under the curve (iAUC) for glucose were calculated.

### 2.3. Real-Time Quantitative Reverse Transcription–Polymerase Chain Reaction (RT-PCR)

RT-PCR was performed to assess the mRNA expression of the insulin signaling pathway molecules Akt2, PI3K, and GLUT4 in the collected skeletal muscle (soleus muscle) using the TaqMan^®^ system according to the manufacturer’s protocols. Skeletal muscles were homogenized, and RNA was extracted using the RNeasy^®^ Mini Kit (cat. no. 74104; QIAGEN N.V., Hilden, Germany). The mRNA expression levels of PI3K, Akt2, and GLUT4 were normalized to those of β-actin. The expression levels of the target genes were calculated using the 2−^ΔΔ^Cq method. Primers and probes for SiC2a4 (GLUT4) (Rn01752377_m1; Applied Biosystems, Foster City, CA, USA), Akt2 (Rn00690901_m1), PiK3cg (Rn01769524_m1), and β-actin (Rn00667869_m1) were used for TaqMan^®^ Gene Expression Assays.

### 2.4. Western Blot Assay

Frozen tissues of skeletal muscles were homogenized in a high-speed vibratory crusher. Proteins were extracted from the precipitate using a radioimmunoprecipitation assay buffer (50 mmol/L Tris-HCl buffer (pH 7.6), 150 mmol/L NaCl, 1% Nonidet^®^ P40, 0.5% sodium deoxycholate, protease inhibitor cocktail, 0.1% sodium dodecyl sulfate) (CAT no. 08714-04; Nacalai Tesque, Kyoto, Japan). Protein concentrations were then quantified using the Pierce™ BCA Protein Assay Kit (CAT no. 23225; Thermo Fisher Scientific, Inc., Danvers, Waltham, MA, USA). The polyvinylidene difluoride (PVDF) membrane was blocked with Bullet Blocking One for western blotting (CAT no. 13779-56; Nacalai Tesque, Kyoto, Japan) for 5 min and then incubated overnight at 5 °C with the following primary antibodies: rabbit anti-Akt2 monoclonal antibody (1:1000; CAT no. 3063s; Cell Signaling Technology, Inc., Danvers, MA, USA), mouse anti-GLUT4 monoclonal antibody (1:1000; CAT no. 2213, Cell Signaling Technology, Inc.), rabbit anti-PI3K p85 monoclonal antibody (1:2000; CAT no. 4257S; Cell Signaling Technology, Inc.), and rabbit anti- glyceraldehyde-3-phosphate dehydrogenase (GAPDH) monoclonal antibody (1:10,000; CAT no. 5174S; Cell Signaling Technology, Inc.), and rabbit anti-GAPDH monoclonal antibody (1:10,000; CAT no. 5174S; Cell Signaling Technology, Inc.). Glyceraldehyde-3-phosphate dehydrogenase was used as an internal reference. The PVDF membrane was washed three times with Tris-buffered saline containing 0.1% Tween-20 (TBST) and was incubated with either horseradish peroxidase (HRP)–conjugated goat anti-rabbit immunoglobulin G (IgG; 1:10,000; CAT no. 7074, Cell Signaling Technology, Inc.) or HRP-conjugated goat anti-mouse IgG (1:10,000; CAT no. 7076, Cell Signaling Technology, Inc.) for 1 h at approximately 25 °C. After washing the PVDF membrane three times with TBST, the blots were developed using an ImmunoStar LD (CAT no. 296-69901; FUJIFILM Wako Pure Chemical Corporation, Osaka, Japan), and the intensity of the bands was quantified using fusion software (ver. 1.51; National Institute of Health, Bethesda, MD, USA).

### 2.5. Immunohistochemistry for Adipophilin

Four-micrometer paraffin-embedded tissue sections were used for staining. The slides were immersed in an antigen retrieval solution consisting of citrate buffer, pH 6.0 and placed in the autoclave at 121 °C for 10 min. Immunohistochemical staining was performed for detection of intracellular lipid using anti-adipophilin antibody (clone AP125, Progen Biotechnik GmbH, Heidelberg, Germany) diluted 1:50. Slides were incubated with primary antibody overnight at 4 °C. A secondary antibody was added for 1 h at 25 °C. The reaction was developed with diaminobenzidine and counterstained with hematoxylin. The positive-stained area of adipophilin was quantitated using Image J software (NIH Image, Bethesda, MD, USA). Three images of liver and skeletal muscle sections from each rat were randomly photographed, and the % of area stained was analyzed.

### 2.6. Statistical Analysis

Results are presented as the mean ± standard deviation. Differences between the two groups were determined using the Mann–Whitney U test. Statistical significance was indicated using *p* < 0.05. Pearson analysis was used for analyzing the association of the insulin signaling and iAUC. All statistical analyses were performed using GraphPad Prism V.8.4.2 (GraphPad Software Inc., San Diego, CA, USA).

## 3. Results

### 3.1. Body Weight

The birth weight difference between FGR and control group rats was significant (*p* < 0.05), with the mean birth weight being 5.8 ± 0.6 g in the FGR group (n = 7) and 6.5 ± 0.2 g in the control group (n = 7). No significant difference was observed in the body weight between the two groups from four weeks of age onwards (*p* > 0.05) (Table 1).

### 3.2. OGTT

**Figure 2:**
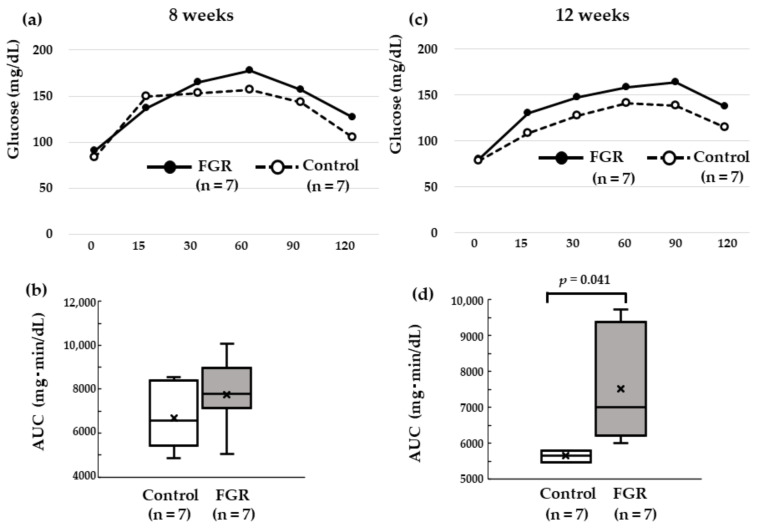
Incremental areas under the curve (iAUC; 0–120 min) of blood glucose: (**a**,**b**) At 8 weeks postnatal, median values of iAUC was 7785 (mg·min/dL) in the FGR group (n = 7) and 6585 (mg·min/dL) in the control group (n = 7) (*p* = 0.19). (**c**,**d**) At 12 weeks postnatal, median values of iAUC was 7012 (mg·min/dL) in the FGR group (n = 7) and 5655 (mg·min/dL) in the control group (n = 7) (*p* = 0.041).

At eight weeks of age, the iAUCs (0–120 min) of blood glucose levels in the two groups were not significantly different (7735 ± 1579 mg·min/dL and 6701 ± 1378 mg·min/dL in FGR and control rats, respectively; *p* > 0.05; Figure 2a,b). At 12 weeks of age, the iAUC (0–120 min) of blood glucose in the FGR group (7520 ± 1298 mg·min/dL) was significantly higher (*p* < 0.05) than that in the control group (5645 ± 617 mg·min/dL; Figure 2c,d).

### 3.3. RT-PCR of Insulin Signaling in Skeletal Muscles

**Figure 3:**
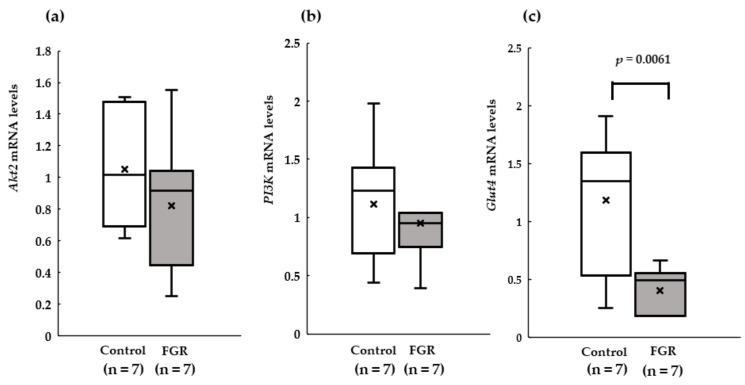
Median values of real-time quantitative reverse transcription–polymerase chain reaction for (**a**) protein kinase B (*Akt2*), (**b**) phosphoinositide 3-kinases (*PI3K*), and (**c**) glucose transporter type 4 (*Glut4*) in the FGR and control groups. The mRNA expression levels of *PI3K*, *Akt2*, and *Glut4* were normalized to those of β-actin.

At 12 weeks of age, *Glut4* mRNA expression in the soleus muscle was significantly lower in the FGR group than that in the control group (*p* < 0.05). The mRNA expression of *PI3K* and *Akt2* did not show any significant difference (*p* > 0.05) (Figure 3).

### 3.4. Western Blot Analysis of Insulin Signaling in Skeletal Muscles

**Figure 4:**
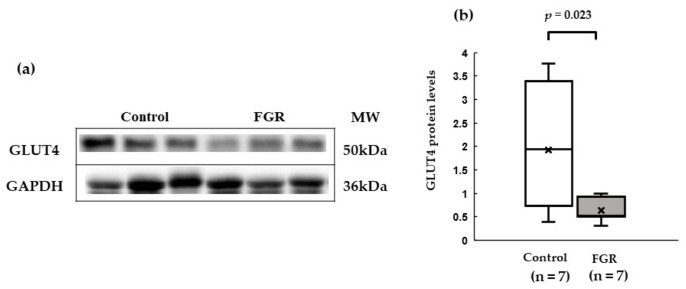
Expression of total GLUT4 protein levels in skeletal muscle tissue. (**a**) Representative western blot images for GLUT4 and GAPDH. (**b**) Median relative expression levels of GLUT4 normalized to those of the housekeeping gene GAPDH. MW: molecular weight.

**Figure 5:**
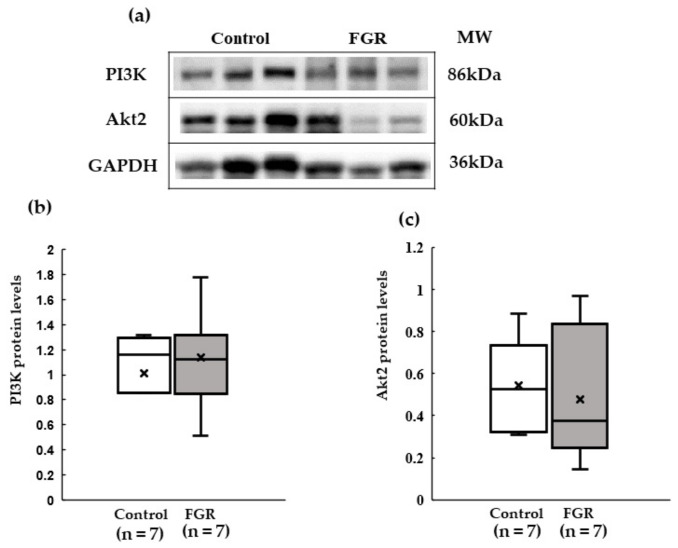
Expression of the protein levels of Akt2 and PI3K in skeletal muscle tissue. (**a**) Representative western blot images for PI3K, Akt2 and GAPDH. (**b**) Median relative expression levels of PI3K normalized to the housekeeping gene GAPDH. (**c**) Median relative expression levels of Akt2 normalized to those of the housekeeping gene GAPDH. MW: molecular weight.

GLUT4 protein expression was significantly lower in the soleus muscle of the FGR group than that in the control group (Figure 4). The expression of the insulin signaling pathway molecules *PI3K* and *Akt2* in the soleus muscle did not differ at the protein level between the two groups (Figure 5).

### 3.5. Association between GLUT4 Expression and iAUC at 12 Weeks of Age

Although not statistically significant, there was a tendency toward a negative correlation between iAUC and the GLUT4 mRNA (r = −0.53, *p* = 0.054) and protein (r = −0.508, *p* = 0.067) expression levels in the soleus muscle at 12 weeks of age.

### 3.6. Intracellular Lipid DROPLET accumulation in the Liver and Skeletal Muscle

**Figure 6:**
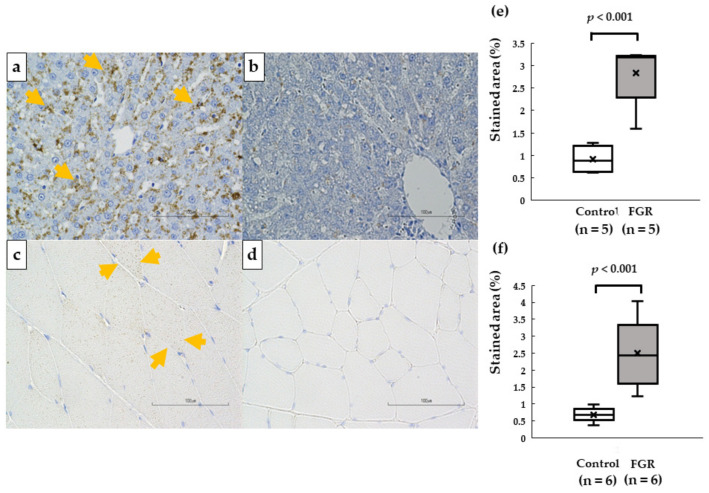
Micrographs of a cross section of the liver and skeletal muscle using immunohistochemistry for adipophilin in a FGR rat ((**a**): liver, (**c**): skeletal muscle) and control rat ((**b**): liver, (**d**): skeletal muscle) at 12 weeks of age. Increased numbers of adipophilin-positive granules in the liver and muscle fibers were observed in FGR rats. Bar = 100 μm. Median values of the percent-stained area of adipophilin in the liver (**e**) and skeletal muscle (**f**).

Small vacuoles upon adipophilin immunohistochemistry of liver cells were observed in the FGR rats and not in the control rats at 12 weeks of age. The numbers of muscle fibers containing vacuoles were also higher in the FGR rats (Figure 6). The percent-stained area of adipophilin was significantly higher in the liver and skeletal muscle of the FGR group than that in the control group (Figure 6).

## 4. Discussion

To the best of our knowledge, this pilot study represents the first report of skeletal muscle insulin resistance in a reduced uterine artery blood flow FGR rat model. As described by Kitase et al. [20], we successfully constructed an FGR rat model, as indicated by the reduced birth weight of the pups.

There was no weight difference between the two groups at 12 weeks of age. Moreover, there was no excessive catch-up or rats that were overweight in the FGR group and the OGTT showed that the iAUC of blood glucose in the FGR group was significantly higher than that in the control group. Therefore, our results indicated that insulin sensitivity was decreased in FGR rats during childhood without obesity. Previously, it had also been reported that placentally restricted FGR rats were similar to FGR humans in terms of decreasing insulin sensitivity and impaired insulin secretion [22]. Furthermore, the development of T2DM at 6 months of age was found in a uterine artery ligation FGR rat model [18,19].

Insulin stimulation is known to induce insulin signaling through receptor-binding in target cells [23,24]. There are two types of fibers (type I fibers and type II fibers) in the muscle that constitutes the lower leg skeletal muscle. Type I fibers have a slow contraction rate and weak muscle strength, but they have markedly high muscle endurance and aerobic metabolic capacity. It has been previously reported that insulin sensitivity correlates with the proportion of type I fibers [25] and that the soleus muscle is primarily composed of type I fibers [26,27]. Therefore, we aimed to elucidate the mechanism of insulin resistance by analyzing the soleus muscle in this study.

Insulin-stimulated glucose transport is greater in skeletal muscle enriched in type I fibers [28], possibly related to a higher GLUT4 content [29,30]. Insulin resistance in muscle tissue is associated with reduced numbers and activity of GLUT4 [31]. Some studies using FGR rats reported a decrease in GLUT4 expression at the protein level in skeletal muscles due to maternal malnutrition [32,33]. Wang et al. [34] also reported that in pigs, offspring born to nutrient-deficient mothers had reduced expression of GLUT4, both during childhood and after growth. There has also been a report of decreased GLUT4 expression in skeletal muscles in fetuses with maternal hypoxemia [35]. In a study on skeletal muscle biopsy specimens from human adults, PI3K and GLUT4 expression was reduced in adults with FGR, despite regular insulin receptor composition [36]. Our results suggest that the reduced expression of GLUT4 in the soleus muscle may play an important role in skeletal muscle insulin resistance in young adults, which is in agreement with past findings. Here, we did not find a decrease in PI3K levels in the soleus muscle; only a decrease in GLUT4 expression was noted. Xing et al. [32] reported that reduction of GLUT4 expression is possibly mediated by decreased PI3K and phosphorylated Akt levels in maternal protein-restricted FGR models. This may be because in the fetal hypoperfusion model, blood flow is reduced from GD-17, a shorter period than that in the hypoprotein–induced FGR model, in which low protein nutrition is administered from the beginning of pregnancy. Gamm et al. [37] reported that muscle protein expression of GLUT4 was decreased in offspring of maternal hypoxic rats, although there was no significant difference in the expression of PI3K. Although the evaluation of the activation/phosphorylation levels of Akt, PI3K, and protein kinase C might shed further light on the potential mechanism underlying insulin resistance in our system, we could not evaluate the activation/phosphorylation levels of these proteins in this study—these experiments were attempted; however, no conclusive results could be drawn from them. Furthermore, we evaluated the Akt2 isoform, which exhibits specificity toward the insulin signaling pathway and a regulatory PI3K subunit (p85) according to previous reports [38,39,40]. We could not analyze other Akt isoforms and catalytic PI3K subunits in this study.

Our results indicated that there may exist alternative mechanisms to determine the reduction of GLUT4 expression independent of the alteration of AKT and PI3K levels. Recently, several microRNAs (miRNAs) and a structural homologue of the E2 ubiquitin-conjugating enzymes have also been suggested as potential regulators of GLUT4 expression. Specifically, increased miR-29b-3p and miR-29c-3p expression correlates with decreased GLUT4 content in the skeletal muscle of diabetic rats [41], and the small ubiquitin-modifier conjugating enzyme 9 has been directly related to GLUT4 content in muscular cells [42]. Further investigation of these regulators in our FGR rat model is therefore warranted.

We also identified intracellular lipid droplets in the FGR rats using immunohistochemistry for adipophilin. Intramyocellular lipid droplets were associated with increased insulin resistance in skeletal muscle fibers [43]. Adipophilin is essential for lipid storage by enhancing the division of excess fatty acids toward triglyceride storage in lipid droplets, leading to lipotoxicity-associated insulin resistance [44]. Furthermore, a recent review indicated that lipotoxicity plays an important role in the development of both insulin resistance and muscle atrophy in patients with type 2 diabetes [45]. It has been previously reported that FGR induces adipose dysfunction [46]. Dysfunctional adipocytes lose their ability to store lipids, inducing a redirection of lipids toward the muscles and liver [47]. Studies in humans and animals have shown that body weight may not reflect body fat storage [48,49], and here, FGR offspring had greater lipid contents in their muscles compared to the control group [50,51]. Therefore, the increase in intracellular lipid droplets in the liver and skeletal muscle was considered to be related to the insulin resistance of the FGR rats.

## 5. Conclusions

Reduced placental blood flow in the AC-attached FGR model rats resulted in skeletal muscle insulin resistance. This was likely due to the decreased expression of some insulin signaling molecules in the soleus muscle. There is thus a possibility of FGR causing insulin resistance in young adulthood without obesity. Therefore, appropriate insulin resistance evaluation and intervention is important in children and young adults with FGR.

## Figures and Tables

**Table 1:** Changes in the body weights of control and fetal growth restriction rats up to 12 weeks of age. Values denote mean ± standard deviation. * *p* = 0.002.

	Control (n = 7)	FGR (n = 7)
**Birth**	6.5 ± 0.2 g	5.8 ± 0.6 g *
**4 weeks**	96.0 ± 13.3 g	84.4 ± 12.6 g
**8 weeks**	315.9 ± 9.8 g	346.3 ± 38.9 g
**12 weeks**	436.1 ± 16.1 g	430.6 ± 51.2 g

## Data Availability

Not applicable.
